# HIV, tuberculosis, diabetes mellitus and hypertension admissions and premature mortality among adults in Uganda from 2011 to 2019: is the tide turning?

**DOI:** 10.1186/s41182-022-00447-y

**Published:** 2022-08-10

**Authors:** Andrew Kazibwe, Kuteesa Ronald Bisaso, Andrew Peter Kyazze, Sandra Ninsiima, Phillip Ssekamatte, Felix Bongomin, Joseph Baruch Baluku, Davis Kibirige, George Patrick Akabwai, Moses R. Kamya, Harriet Mayanja-Kizza, Pauline Byakika-Kibwika, Magid Kagimu, Robert Kalyesubula, Irene Andia-Biraro

**Affiliations:** 1 Department of Internal Medicine, School of Medicine, Makerere University College of Health Sciences, P. O. Box 7072, Kampala, Uganda; 2 Directorate of Programs, The AIDS Support Organisation, P. O. Box 10443, Kampala, Uganda; 3 Breakthrough Analytics, Kampala, Uganda; 4 Tuberculosis and Co-Morbidities (TAC) Research Group, Makerere University, Kampala, Uganda; 5 Department of Immunology and Molecular Biology, School of Biomedical Sciences, Makerere University College of Health Sciences, P. O. Box 7072, Kampala, Uganda; 6 Department of Medical Microbiology and Immunology, Faculty of Medicine, Gulu University, P. O. Box 166, Gulu, Uganda; 7 Division of Pulmonology, Kiruddu National Referral Hospital, Kampala, Uganda; 8 Makerere University Lung Institute, Makerere University College of Health Sciences, Kampala, Uganda; 9 Uganda Martyrs Hospital, Lubaga, P. O. Box 14130, Kampala, Uganda; 10 Baylor College of Medicine, Children’s Foundation, Kampala, Uganda; 11 Department of Physiology, School of Biomedical Sciences, Makerere University College of Health Sciences, Kampala, Uganda; 12 Medical Research Council/Uganda Virus Research Institute and London School of Hygiene and Tropical Medicine Uganda Research Unit, Entebbe, Uganda

**Keywords:** Trend, Premature mortality, Inpatient, HIV, Tuberculosis, Diabetes mellitus, Hypertension, Uganda

## Abstract

**Background:**

The growing burden of diabetes mellitus (DM) and hypertension (HTN) on the background of endemic Human Immuno-deficiency Virus (HIV) and tuberculosis (TB) is a concern in low- and middle-income countries. We aimed to describe annual trends in admissions, mortality rates and premature mortality (years of potential life lost—YPLLs) due to HIV, tuberculosis (TB), diabetes mellitus (DM) and hypertension (HTN) in Uganda.

**Methods:**

We conducted a retrospective cohort study, retrieving electronic records of adults admitted to Mulago and Kiruddu national referral hospitals medical wards between 1st January 2011 and 31st December 2019. We used STATA BE 17.0 and GraphPad Prism 8.0.2 to compute total admissions, inpatient crude mortality rates, and YPLLs; and demonstrate trends using Mann–Kendall test.

**Results:**

Of 108,357 admissions, 55,620 (51.3%) were female, 15,300 (14.1%) were recorded in 2012, and 22,997 (21.2%) were aged 21–30 years. HIV, TB, DM and HTN accounted for 26,021 (24.0%); 9537 (8.8%); 13,708 (12.7) and 13,252 (12.2%) of all admissions, respectively. Overall inpatient mortality was 16.7% (18,099/108,357), 53.5% (9674/18,099) were male, 21.5% (3898) were aged 31–40 years and 2597 (14.4%) were registered in 2013. HIV, TB, DM and HTN accounted for 35.6% (6444), 14.6% (2646), 9.1% (1648) and 11.8% (2142) of all deaths, respectively. Total admissions (Kendall’s tau-B = − 0.833, *p* < 0.001) and deaths declined (Kendall’s tau-B = − 0.611, *p* = 0.029). A total of 355,514 (mean = 20.8 years, SD 30.0) YPLLs were recorded, of which 54.6% (191,869) were in males; 36.2% (128,755) were among those aged 21–30 years and were recorded in 2012 (54,717; 15.4%). HIV, TB, DM and HTN accounted for 46.5% (165,352); 19.5% (69,347); 4.8% (16,991) and 4.5% (16,167) of YPLLs, respectively. Proportionate contribution of HIV to deaths and YPLLs declined, remained stagnant for TB; and increased for both DM and HTN.

**Conclusion:**

TB and HIV account for higher though declining, while DM and HTN account for lower albeit rising morbidity and premature mortality among adult medical patients in Uganda. TB prevention and treatment; and DM/HTN service integration in HIV care should be optimized and scaled up.

## Introduction

Human immunodeficiency virus (HIV) and tuberculosis (TB) still account for substantial adult morbidity and mortality in low- and middle-income countries (LMICs), Uganda inclusive [1]. In 2020, there were over 7000 TB-related deaths and 90,000 new TB cases in Uganda [2]. Moreover, there were 38,000 new HIV infections and 21,000 HIV-related deaths in Uganda in 2020 [3]. There is, however, growing concern over the rapidly rising burden of premature mortality attributable to non-communicable diseases (NCDs) such as diabetes mellitus (DM) and hypertension, in Uganda and sub-Saharan Africa [4]. The rise in DM is expected to retard progress towards elimination of TB since it increases the risk of active TB disease threefold, and increases risk of sub-optimal TB treatment outcomes [5]. In addition, the scale-up of universal ART has resulted in more people living with HIV (PLHIV) surviving into older age, and hypertension, a key risk factor for cardiovascular mortality has been identified as a leading cause of non-AIDS-related mortality among PLHIV [6]. The dual burden of NCDs and infectious diseases is expected to strain health systems in LMICs and Uganda in particular [7–9].

Due to the rapid scale-up of universal antiretroviral therapy in Uganda, over the last nearly two decades, HIV incidence, morbidity and mortality have declined [10, 11]. However, from a recent study of adult patients admitted at a national referral hospital, at least 25% of admissions were HIV positive and HIV-positive individuals contributed almost half of all deaths [12]. Moreover, the same study showed a rise in inpatient mortality over a period of 4 years [12]. This was on the background of increasing proportions of patients admitted with hypertension and diabetes mellitus [12]. It however remains unclear whether these observations can be attributed to the growing burden of NCD-associated morbidity among ageing PLHIV or a growing burden of HIV among young people with NCDs. To inform re-prioritization of resources for age-appropriate NCD or HIV disease prevention, assessment of trends in premature mortality attributable to each disease condition is important. The Years of Potential Life Lost (YPLL) metric, introduced by the United States Centers for Disease Control and Prevention (CDC) in 1982, overcomes the effect of ageing-related disease deaths that often skew relative mortality measures, by weighting each death as the difference between an expected age of survival and observed age at death [13–17]. This allows for distinction between true increase in disease-specific mortality and increase in mortality attributable to ageing.

In this study, we determined disease (HIV, TB, DM and hypertension) and sex-specific trends in proportionate admissions, mortality, and premature mortality (YPLL) on adult medical inpatient wards at Mulago and Kiruddu national referral hospitals in Uganda over a 9-year-period (2011–2019).

## Methods

### Study design

This was a retrospective cohort study. We obtained patient data from the Rainer Arnolds Senior House Officers Training support (RASHOTs) database for patients admitted to adult medical inpatient wards at Mulago and Kiruddu national referral hospitals (NRH) from 2011 to 2019 [12].

### Study setting

We retrieved records of patients admitted to the adult medical wards at Mulago NRH (2011–2014) and Kiruddu NRH from 2014 to 2020. In 2014, the Government of Uganda (GoU) undertook a major renovation of Mulago NRH that necessitated migration of medical inpatient services to Kiruddu Hospital, which had been newly upgraded from a divisional hospital [18, 19]. Mulago NRH is located in Kawempe Division; whereas Kiruddu NRH is located in Makindye division of the capital city. The RASHOTs database was the central repository for adult medical inpatient data for the study period.

### Data collection

We retrieved data on patient sex, age at admission, and diagnoses at discharge or death; and downloaded as spreadsheets, which we cleaned and conducted preliminary analyses using Microsoft Excel. Diagnoses were made by specialist physicians on inpatient wards using clinical algorithms involving patient clinical history, physical examination, laboratory tests and radiological imaging, where applicable and available. We carried out additional analyses and generated graphs using GraphPad Prism 8. Individuals with incomplete data (age, sex, or diagnoses) were excluded from the data analysis. For each patient who died, we computed YPLL by subtracting the age at death from the sex-specific life expectancy at birth in Uganda as determined by the 2014 Population Census, i.e. female—64.5 years and male—62.8 years [20]. Where the difference was less than zero (patient died at an age older than the sex-specific life-expectancy), the YPLL was reported as zero.

### Data analysis

We determined percentage contribution to total inpatient admissions**,** mortality and YPLLs for each sex, age group and major disease conditions: HIV, TB, DM, and Hypertension. We summarized patient characteristics as frequencies and demonstrated trends using line graphs; and, computed total and mean YPLLs by year, sex, and disease condition**.** We calculated inpatient mortality rate as a percentage of deaths divided by number of admissions. We determined disease, age-group and sex-specific total annual YPLLs and used Mann–Kendall test to analyse for trends in number of admissions, deaths and total annual YPLLs. We applied the Mann–Kendall test to overall absolute numbers of admissions, deaths and total YPLLs; and then to proportions of the different sub-populations; using Stata BE 17.0. We compared median YPLLs between sexes and disease classes using Mann–Whitney *U* test. A *p* < 0.05 was considered statistically significant. We defined trend as the magnitude and direction of the Mann–Kendall tau-B coefficient between the outcome and time in years (2011–2019).

## Results

**Fig. 1:**
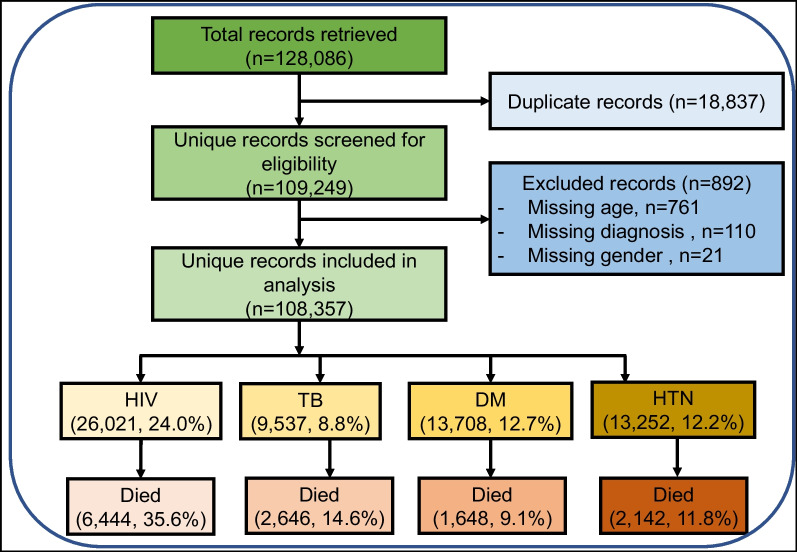
Study flow diagram and patient summary

We retrieved 128,086 records, of which 18,837 were duplicates, and 892 were excluded because of incomplete data as shown in Fig. 1.

### Trends in admissions

Of a total of 108,357 inpatient admissions, 51.3% (55,620) were female, 21.2% (22,997) were aged 21–30 years, 14.1% (15,300), 24.0% were HIV positive. The proportion of admissions that were 41–50, 51–60 and over 70 years of age increased across the period (Table 1).

**Table 1:** Annual trends in total admissions by sex, age group and disease condition

	Overall	2011	2012	2013	2014	2015	2016	2017	2018	2019	Kendall’s tau-B (*p*-value, Kendall’s score, SE)
Total (row %)	**108,357**	**13,787 (12.7)**	**15,300 (14.1)**	**14,622 (13.5)**	**13,795 (12.7)**	**13,289 (12.3)**	**10,477 (9.7)**	**10,229 (9.4)**	**9163 (8.5)**	**7695 (7.1)**	− **0.83 (< 0.01, **− **30, 9.59)**
Sex											
Female (%)	55,620 (51.3)	7038 (51.0)	7913 (51.7)	7518 (51.4)	7181 (52.1)	6850 (51.5)	5441 (51.9)	5109 (49.9)	4620 (50.4)	3950 (51.3)	− 0.17 (0.60, − 6, **9.59**)
Age mean (SD)	43.3 (19.4)	41.8 (18.9)	41.4 (18.8)	42.5 (19.3)	43.8 (19.4)	43.5 (19.6)	43.5 (19.8)	43.8 (19.6)	44.6 (19.6)	45.4 (19.5)	
20 years and below (%)	**12,550 (11.6)**	**1751 (12.7)**	**1902 (12.4)**	**1746 (11.9)**	**1457 (10.6)**	**1533 (11.5)**	**1265 (12.1)**	**1230 (12.0)**	**957 (10.4)**	**709 (9.2)**	− **0.61 (0.03, **− **22, 9.59)**
21–30 years (%)	**22,997 (21.2)**	**3136 (22.7)**	**3563 (23.3)**	**3216 (22.0)**	**2896 (21.0)**	**2829 (21.3)**	**2177 (20.8)**	**2003 (19.6)**	**1739 (19.0)**	**1438 (18.7)**	− **0.89 (< 0.01, **− **22, 9.59)**
31–40 years (%)	**21,362 (19.7)**	**2818 (20.4)**	**3178 (20.8)**	**2971 (20.3)**	**2734 (19.8)**	**2564 (19.3)**	**1969 (18.8)**	**1881 (18.4)**	**1826 (19.9)**	**1421 (18.5)**	− **0.67 (0.02, **− **24, 9.59)**
41–50 years (%)	16,812 (15.5)	2056 (14.9)	2393 (15.6)	2246 (15.4)	2207 (16.0)	2010 (15.1)	1615 (15.4)	1650 (16.1)	1380 (15.1)	1255 (16.3)	0.33 (0.25, 12, 9.59)
51–60 years (%)	**12,904 (11.9)**	**1495 (10.8)**	**1595 (10.4)**	**1635 (11.2)**	**1682 (12.2)**	**1626 (12.2)**	**1196 (11.4)**	**1323 (12.9)**	**1246 (13.6)**	**1106 (14.4)**	**0.83 (< 0.01, 30, 9.59)**
61–70 years (%)	**10,288 (9.5)**	**1304 (9.5)**	**1317 (8.6)**	**1334 (9.1)**	**1285 (9.3)**	**1245 (9.4)**	**1053 (10.1)**	**992 (9.7)**	**937 (10.2)**	**821 (10.7)**	**0.72 (0.01, 26, 9.59)**
70 + years (%)	**11,444 (10.6)**	**1227 (8.9)**	**1352 (8.8)**	**1474 (10.1)**	**1534 (11.1)**	**1482 (11.2)**	**1202 (11.5)**	**1150 (11.2)**	**1078 (11.8)**	**945 (12.3)**	**0.89 (< 0.01, 32, 9.59)**
HIV +^a^ (%)	**26,021 (24.0)**	**3447 (25.0)**	**4244 (27.7)**	**3956 (27.1)**	**3432 (24.9)**	**2785 (21.0)**	**2312 (22.1)**	**2289 (22.4)**	**1990 (21.7)**	**1566 (20.4)**	− **0.67 (0.02, **− **24, 9.59)**
TB^b^ (%)	9537 (8.8)	1516 (11.0)	1874 (12.2)	1494 (10.2)	1087 (7.9)	846 (6.4)	674 (6.4)	725 (7.1)	732 (8.0)	589 (7.7)	− 0.39 (0.178, − 14, **9.59**)
DM^c^ (%)	13,708 (12.7)	1748 (12.7)	1780 (11.6)	1897 (13.0)	1807 (13.1)	1672 (12.6)	1362 (13.0)	1162 (11.4)	1202 (13.1)	1078 (14.0)	0.39 (0.18, 14, **9.59**)
HTN^d^ (%)	13,252 (12.2)	1074 (7.8)	1493 (9.8)	1620 (11.1)	1921 (13.9)	1824 (13.7)	1429 (13.6)	1280 (12.5)	1321 (14.4)	1290 (16.8)	0.67 (0.02, 24, **9.59**)

^a^HIV: immunosuppression syndrome, advanced HIV disease, HIV

^b^Tuberculosis: all forms—pulmonary, extrapulmonary including TB meningitis (TBM) and TB sepsis

^c^Diabetes mellitus, diabetic ketoacidosis (DKA), diabetic nephropathy, diabetic coma, diabetic foot, diabetic neuropathy

^d^Hypertension, hypertensive encephalopathy, hypertensive kidney disease, high blood pressure, hypertensive heart disease (HHD)

SE = standard error of score

Bold—statistically significant tau-B value indicating a significant trend

Among females, HIV or TB admissions were highest in the 21–30 years age group (34.5%, 40.1%, respectively) while among males, it was in the 31–40 years age group (37.2% and 35.4%, respectively). DM admissions among females were highest in the 51–60 age group (24.2%), and in the 41–50 years age group among males (20.3%). HTN admissions among both females and males were highest in those above 70 years age group (24.5% and 21.6%, respectively).

**Fig. 2:**
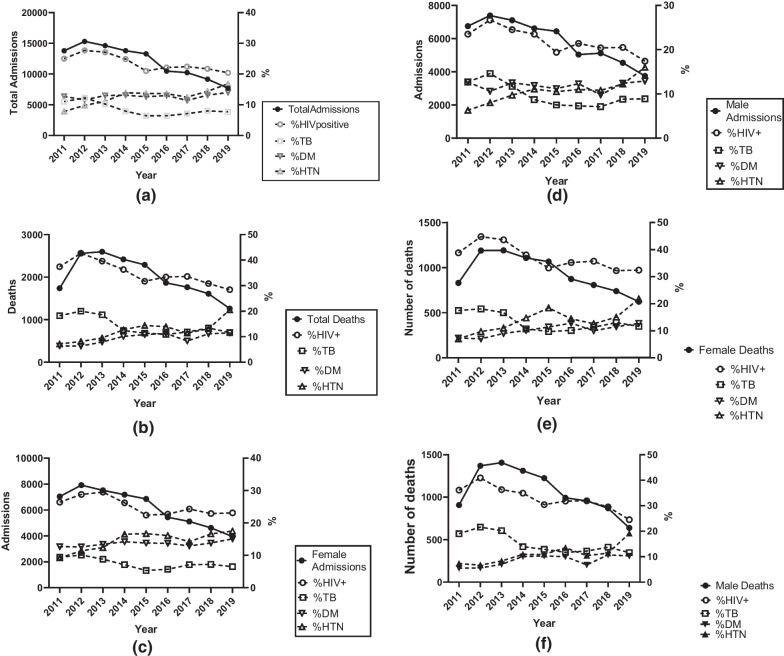
Annual trends in total and proportionate admissions and deaths by disease. **a** Annual trends in total and proportionate admissions by disease. **b** Annual trends in female total and proportionate admissions by disease. **c** Annual trends in male total and proportionate admissions by disease. **d** Annual trends in total and proportionate deaths by disease. **e** Annual trends in female total and proportionate deaths by disease. **f** Annual trends in male total and proportionate deaths by disease

Total admissions declined (T_B_ = − 0.83; *p* < 0.01, Kendall’s score = − 30, SE = 9.59); the proportions of admissions in the age groups: 20 years and below; 21–30 years, 31–40 years declined (T_B_ = − 0.61, *p* = 0.03, score = −22; T_B_ = − 0.89, *p* < 0.01, Kendall’s score = − 22; T_B_ = − 0.67, *p* = 0.02, Kendall’s score = − 24, SE = 9.59, respectively) while the proportions of admissions in the older age groups (51–60, 61–70 and over 70 years) increased. The proportions of patients admitted with HIV declined (T_B_ = − 0.67; *p* = 0.02, Kendall’s score = − 24, SE = 9.59), remained stable for TB (T_B_ = − 0.39, *p* = 0.18, Kendall’s score = − 14) and DM (T_B_ = 0.39, *p* = 0.18, Kendall’s score = 14, SE = 9.59) while these increased for HTN (T_B_ = 0.67, *p* = 0.02, Kendall’s score = 24 SE = 9.59) (Table 1, Fig. 2a) and these appeared similar between males and females (Fig. 2b, c).

### Comorbidity among admissions

Among HIV admissions, 25.9% (6738/26,021) of HIV-positive patients had tuberculosis, 2.6% (666) and 2.6% (665) had diabetes mellitus and hypertension, respectively. Among patients with tuberculosis, 70.7% (6738/9537) had HIV, 2.2% (214) and 1.0% (94) had diabetes mellitus and hypertension, respectively. Among patients with diabetes mellitus, 4.9% (666/13708), 1.6% (214), 27.6% (3790) had HIV, tuberculosis and hypertension, respectively. In patients with hypertension, 5% (665/13252), 0.7% (94), and 28.6% (3790) had HIV, TB and DM, respectively.

### Trends in inpatient mortality rate

Overall inpatient mortality rate of 16.7% (18,099 deaths) was registered. Overall absolute number of deaths declined (T_B_ = − 0.61, *p* = 0.03, Kendall’s score = − 22, SE of score = 9.59), but inpatient mortality rate remained stable (T_B_ = 0.17, *p* = 0.60, Kendall’s score = 6, SE = 9.59) (Table 2, Fig. 2d–f). Females accounted for 46.5% of deaths, while 35.6% and 24.9% of deaths occurred among HIV-positive patients and ages 31–40 years, respectively. Of the 6444 deaths among HIV-positive patients, 32.1% (2070), 1.7% (108), 2.0% (129) had tuberculosis, diabetes mellitus, hypertension, respectively. Of the 2646 deaths among TB patients, 78.2% (2070), 1.4% (36), 0.6% (17) had HIV, DM or HTN, respectively. Of the 1648 deaths among DM patients, 6.6% (108), 2.2% (36), 33.6% (554) had HIV, TB or HTN, respectively. Of the 2142 deaths among HTN patients, 6.0% (129), 0.8% (17), 25.9% (554) had HIV, TB or HTN, respectively.

**Table 2:** Annual trends in total deaths and proportionate mortality by sex, age group and disease condition

	Overall	2011	2012	2013	2014	2015	2016	2017	2018	2019	Kendall’s tau-B (*p*-value, Kendall’s score, SE)
Total deaths (%)	**18,099**	1737 (9.6)	2558 (14.1)	2597 (14.4)	2418 (13.4)	2290 (12.7)	1866 (10.3)	1763 (9.7)	1609 (8.9)	1261 (7.0)	− **0.61 (0.03, **− **22, 9.59)**
Mortality rate (%)	**16.7**	12.6	16.7	17.8	17.5	17.2	17.8	17.2	17.6	16.4	0.17 (0.60, 6, 9.59)
Sex											
Female (%)	8425 (46.5)	830(47.8)	1189 (46.5)	1191 (45.9)	1107 (45.8)	1066 (46.6)	873 (46.8)	807 (45.8)	739 (45.9)	623 (49.4)	0.00 (> 0.99, 0, 9.59)
Age mean (STD)	46.2 (19.9)	44.3 (19.0)	43.8 (19.2)	44.8 (19.5)	46.4 (20.0)	46.5 (19.5)	47.4 (20.9)	46.4 (19.8)	48.2 (20.2)	49.9 (21.1)	N/A
20 years and below (%)	**1312 (7.2)**	**140 (8.1)**	**206 (8.1)**	**203 (7.8)**	**164 (6.8)**	**152 (6.6)**	**144 (7.7)**	**135 (7.7)**	**92 (5.7)**	**76 (6.0)**	− **0.72 (0.01, **− **26, 9.59)**
21–30 years (%)	**3551 (19.6)**	**357 (20.6)**	**571 (22.3)**	**548 (21.1)**	**479 (19.8)**	**440 (19.2)**	**349 (18.7)**	**323 (18.3)**	**271 (16.8)**	**213 (16.9)**	− **0.83 (< 0.01, **− **30, 9.59)**
31–40 years (%)	**3898 (21.5)**	**432 (24.9)**	**584 (22.8)**	**583 (22.4)**	**524 (21.7)**	**475 (20.7)**	**368 (19.7)**	**358 (20.3)**	**345 (21.4)**	**229 (18.2)**	− **0.78 (< 0.01, **− **28, 9.59)**
41–50 years (%)	2855 (15.8)	251 (14.5)	423 (16.5)	407 (15.7)	389 (16.1)	365 (15.9)	297 (15.9)	283 (16.1)	257 (16.0)	183 (14.5)	− 0.06 (0.92, − 2, 9.59)
51–60 years (%)	2070 (11.4)	181 (10.4)	257 (10.0)	287 (11.1)	264 (10.9)	304 (13.3)	194 (10.4)	217 (12.3)	203 (12.6)	163 (12.9)	0.50 (0.08, 18, 9.59)
61–70 years (%)	**1829 (10.1)**	**178 (10.2)**	**211 (8.2)**	**243 (9.4)**	**235 (9.7)**	**244 (10.7)**	**193 (10.3)**	**202 (11.5)**	**182 (11.3)**	**141 (11.2)**	**0.61 (0.03, 22, 9.59)**
70 + years (%)	**2584 (14.3)**	**198 (11.4)**	**306 (12.0)**	**326 (12.6)**	**363 (15.0)**	**310 (13.5)**	**321 (17.2)**	**245 (13.9)**	**259 (16.1)**	**256 (20.3)**	**0.78 (< 0.01, 28, 9.59)**
HIV + (%)	**6444 (35.6)**	**649 (37.4)**	**1093 (42.7)**	**1029 (39.6)**	**878 (36.3)**	**726 (31.7)**	**623 (33.4)**	**593 (33.6)**	**495 (30.8)**	**358 (28.4)**	− **0.72 (< 0.01, **− **26, 9.59)**
TB (%)	2646 (14.6)	317 (18.2)	511 (20.0)	483 (18.6)	301 (12.4)	261 (11.4)	204 (10.9)	208 (11.8)	215 (13.4)	146 (11.6)	− 0.44 (0.12, − 16, 9.59)
DM (%)	**1648 (9.1)**	**111 (6.4)**	**160 (6.3)**	**205 (7.9)**	**244 (10.1)**	**248 (10.8)**	**211 (11.3)**	**146 (8.3)**	**178 (11.1)**	**145 (11.5)**	**0.72 (< 0.01, 26, 9.59)**
HTN (%)	**2142 (11.8)**	**124 (7.1)**	**207 (8.1)**	**247 (9.5)**	**306 (12.7)**	**329 (14.4)**	**260 (13.9)**	**201 (11.4)**	**210 (13.1)**	**258 (20.5)**	**0.67 (0.02, 24, 9.59)**

Bold—statistically significant tau-B value indicating a significant trend

*SE* standard error of score

Among HIV-positive deaths, majority were in the 31–40 age group (2257/6444, 35.0%). The same age group accounted for the highest proportion of TB deaths (909/2646, 34.4%). Individuals above 70 years of age accounted for most of the deaths in hypertension (703/2142, 32.8%). The proportionate contribution to deaths reduced for the younger age groups and increased for the older age groups.

### Trends in premature mortality

**Fig. 3:**
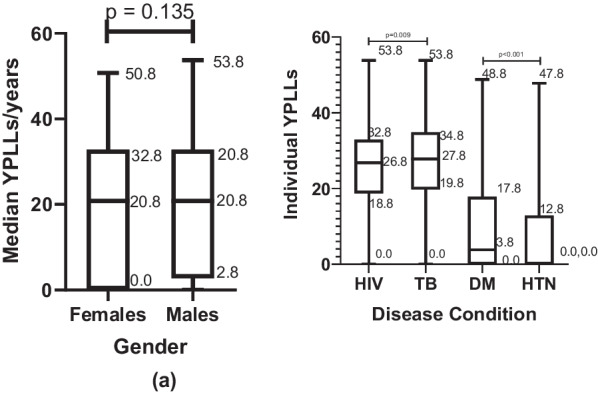
Boxplots comparing overall median YPLLs. **a**. Boxplot comparing overall median YPLLs between males and females. **b**. Boxplot comparing overall median YPLLs among disease conditions

**Fig. 4:**
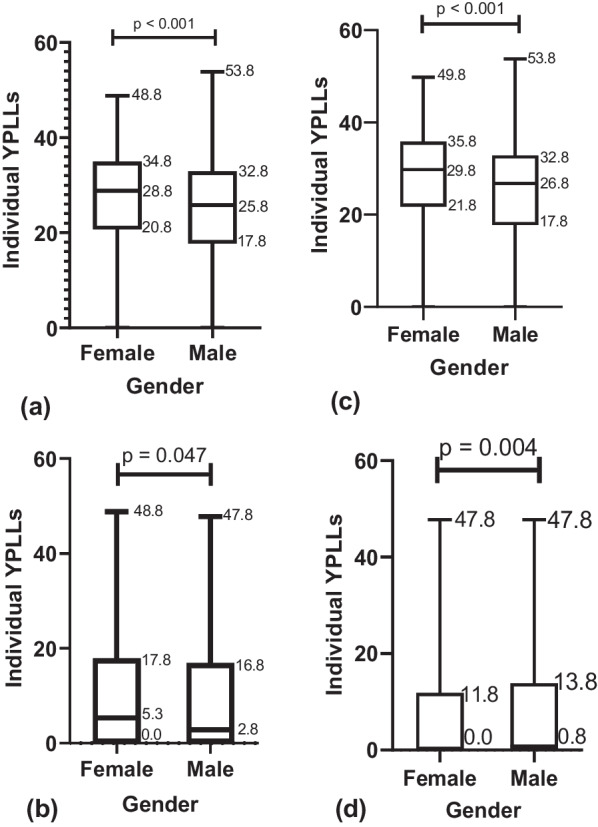
Boxplots comparing disease-specific median YPLLs by sex. **a**. Boxplot comparing median HIV YPLLs between females and males. **b**. Boxplot comparing median DM YPLLs between females and males. **c**. Boxplot comparing median TB YPLLs between females and males. **d**. Boxplot comparing median HTN YPLLs between females and males

The deaths recorded in the period resulted in 355,514 YPLLs; majority of which occurred among males (54.0%), were attributable to HIV (46.5%) and among 21- to 30-year-olds (36.2%) (Table 3). Overall, TB accounted for 19.5% (69,347/355,514) of YPLLs while DM and HTN accounted for 4.8% (16,991) and 4.5% (16,167), respectively. Median YPLL was similar between sexes (20.8, *p* = 0.14) (Fig. 3a). Disease-specific median YPLLs were higher for HIV compared to TB, and higher for HTN compared to DM (Fig. 3b). Among HIV deaths, median YPLL was higher among females than males (27.8 vs 24.8, *p* < 0.01), similarly among TB deaths (29.8 vs 26.8, *p* < 0.001) and DM deaths (5.8 vs 3.8, *p* = 0.08). In HTN deaths, median YPLL was higher among males than among females (11.8 vs 14.8, *p* ≤ 0.01) (Fig. 4).

**Table 3:** Annual trends in total and median YPLLS, and proportion of annual YPLLs of females and age groups

	Overall	2011	2012	2013	2014	2015	2016	2017	2018	2019	Kendall’s tau-B (*p*-value, Kendall’s score, SE)
Total YPLLS	**355,514**	**36,153 (10.2)**	**54,717 (15.4)**	**53,612 (15.1)**	**47,179 (13.3)**	**43,763 (12.3)**	**35,471 (10.0)**	**33,982 (9.6)**	**29,108 (8.2)**	**21,529 (6.1)**	**− 0.78 (< 0.01, − 28, 9.59)**
Mean (SD)	20.8 (30.0)	22.8 (30.0)	22.8 (26.0)	22.8 (29.0)	20.8 (30.0)	19.8 (30.0)	19.8 (32.8)	20.8 (31.0)	17.8 (30.8)	15.8 (30.8)	N/A
Sex											
Female (%)	163,645 (46.0)	17,905 (49.5)	25,257 (46.2)	24,417 (45.5)	21,275 (45.1)	19,089 (43.6)	16,076 (45.3)	16,028 (47.2)	13,097 (45.0)	10,502 (48.8)	− 0.17 (0.60, − 6, 9.59)
Age											
20 years and below (%)	59,990 (16.9)	6393 (17.7)	9473 (17.3)	9289 (17.3)	7515 (15.9)	6952 (15.9)	6610 (18.6)	6138 (18.1)	4165 (14.3)	3455 (16.1)	− 0.22 (0.47, − 26, 9.59)
21–30 years (%)	**128,755 (36.2)**	**12,874 (35.6)**	**20,614 (37.7)**	**19,925 (37.2)**	**17,379 (36.8)**	**15,924 (36.4)**	**12,616 (35.6)**	**11,774 (34.7)**	**9872 (33.9)**	**7776 (36.1)**	− **0.56 (0.05, **− **28, 9.59)**
31–40 years (%)	104,372 (29.4)	11,472 (31.7)	15,606 (28.5)	15,642 (29.2)	14,020 (29.7)	12,650 (28.9)	9916 (27.9)	9582 (28.2)	9300 (32.0)	6183 (28.7)	− 0.17 (0.60, − 26, 9.59)
41–50 years (%)	**48,380 (13.6)**	**4248 (11.8)**	**7268 (13.3)**	**6821 (12.7)**	**6540 (13.9)**	**6157 (14.1)**	**4977 (14.0)**	**4959 (14.6)**	**4387 (15.1)**	**3023 (14.0)**	**0.72 (< 0.01, **− **22, 9.59)**
51–60 years (%)	**13,755 (3.9)**	**1140 (3.2)**	**1729 (3.2)**	**1914 (3.6)**	**1708 (3.6)**	**2044 (4.7)**	**1303 (3.7)**	**1487 (4.4)**	**1363 (4.7)**	**1067 (5.0)**	**0.89 (< 0.01, **− **10, 9.59)**
61–70 years (%)	263 (0.1)	27 (0.1)	27 (0.1)	21 (0.0)	16 (0.0)	36 (0.1)	49 (0.1)	41 (0.1)	22 (0.1)	24 (0.1)	0.28 (0.35, 4, 9.59)
HIV (%)	**165,352 (46.5)**	**17,481 (48.4)**	**28,739 (52.5)**	**26,522 (49.5)**	**22,569 (47.8)**	**18,415 (42.1)**	**15,881 (44.8)**	**15,182 (44.7)**	**12,097 (41.6)**	**8466 (39.3)**	− **0.78 (< 0.01, **− **28, 9.59)**
TB	69,347 (19.5)	8313 (23.0)	13,666 (25.0)	12,518 (23.3)	7860 (16.7)	6711 (15.3)	5535 (15.6)	5481 (16.1)	5614 (19.3)	3648 (16.9)	− 0.28 (0.35, − 10, 9.59)
DM	**16,991 (4.8)**	**897 (2.5)**	**1651 (3.0)**	**1523 (2.8)**	**2397 (5.1)**	**3341 (7.6)**	**2225 (6.3)**	**1614 (4.7)**	**1960 (6.7)**	**1383 (6.4)**	**0.56 (0.05, 20, 9.59)**
HTN	**16,167 (4.5)**	**923 (2.6)**	**1696 (3.1)**	**2184 (4.1)**	**1791 (3.8)**	**2605 (6.0)**	**1594 (4.5)**	**1647 (4.8)**	**1926 (6.6)**	**1801 (8.4)**	**0.83 (< 0.01, 30, 9.59)**

Bold—statistically significant tau-B value indicating a significant trend

*SE* standard error of score

**Fig. 5:**
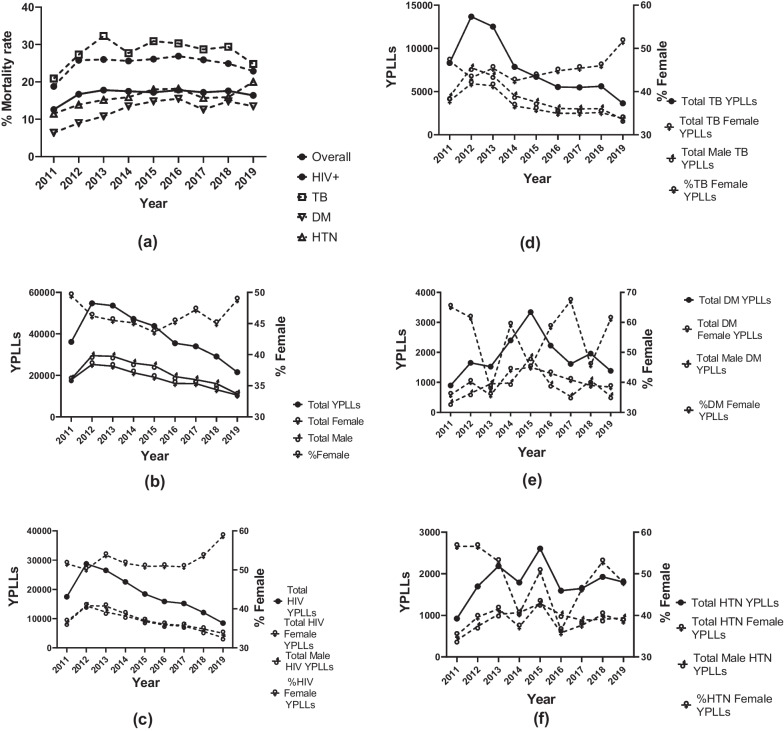
Annual trends in mortality rates, overall and proportionate disease premature mortality by sex. **a**. Annual trends in overall mortality rates by disease. **b**. Annual trends in total and proportionate YPLLs by disease and sex. **c**. Annual trends in HIV YPLLs by sex. **d**. Annual trends in TB YPLLs by sex. **e**. Annual trends in DM YPLLs by sex. **f**. Annual trends in HTN YPLLs by sex

Overall, total YPLLs declined over the decade (T_B_ = − 0.778, *p* < 0.01) (Table 3, Fig. 5b–f).

Proportionate contribution to YPLLs by females remained constant. Over the period, the contribution to YPLLs by HIV declined (T_B_ = − 0.78, *p* < 0.01), remained stable for TB (T_B_ = − 0.28, *p* = 0.35); and increased for both DM and HTN (T_B_ = 0.56, *p* = 0.05; T_B_ = 0.83, *p* < 0.01).

## Discussion

We retrospectively reviewed adult inpatient hospital admission data at national referral hospitals for the period 2011–2019, and analysed for trends in admissions, deaths, mortality rates and premature mortality (measured as years of potential life lost—YPLL), focusing on the two commonest infectious (HIV and TB) and non-infectious (hypertension and diabetes mellitus) disease conditions. More than two-thirds of TB admissions were HIV co-infected, over a quarter of DM patients had hypertension. Females accounted for more than half of HIV-related admissions across the decade. We found declines in overall admissions, deaths, and premature mortality among patients admitted to medical wards at Mulago and Kiruddu NRHs. HIV and TB accounted for the largest proportion of admissions, deaths and YPLLs. Whereas females accounted for more admissions, deaths and YPLLs were higher among males, and yet HIV-related YPLLs were higher among females. Majority of HIV and TB-related admissions and deaths occurred in young adults (21–30 and 31–40 years), while majority of DM and HTN-related admissions and deaths occurred in older individuals. HIV co-morbidity was high among TB patients and TB-related deaths. HIV-, TB- and DM-related YPLLs were higher among females than among males, while hypertension-related YPLLs were higher among males. Proportions of DM and HTN among admissions, deaths and YPLLs increased significantly during the decade.

The decline in patient admissions and absolute number of deaths is probably due to the declining national burden of HIV, which accounted for a significant number of medical inpatient admissions and deaths in the 1990s and early 2000s [21, 22]. In 1994, Tembo et al. reported that HIV accounted for 55.6% of adult inpatient admissions at a large urban hospital in Uganda [21]. In addition, the total years of life lost among admitted medical patients, due to endemic diseases such as HIV and TB is declining over the years [23], in tandem with reported declines in HIV-associated morbidity and mortality over the last decade due to roll-out of universal antiretroviral therapy [11, 24–26]. Our study also found that HIV-related conditions still account for the highest proportion of premature mortality in this population. This is similar to what was found by Rumisha et al. in Tanzania where HIV was the leading cause of premature mortality [27], and is further supported findings from the Global Burden of Disease study where HIV is among the leading causes of mortality in Uganda [1, 28]. In an earlier autopsy study describing causes of death among hospitalized patients at Mulago NRH, 83% of the deaths among HIV + individuals were from infectious causes [29]. Equally, between 2002 and 2012, at an urban HIV clinic, TB and other infectious diseases accounted for majority of deaths. Over the period, the contribution of TB and other infectious diseases declined, whereas non-communicable disease conditions accounted for a growing number of deaths in this population [30]. In a similar study in Malawi, 71% of deaths among patients less than 55 years of age were due to infectious diseases [31], while another study in Southern Nigeria also showed infectious diseases as a leading cause of inpatient mortality [32].

The higher burden of premature mortality attributable to HIV among females than males corroborates the higher prevalence of HIV among females at younger age in Uganda, and is also supported by global and local reports that show a rising number of new HIV infections among adolescent girls and young women [33, 34]. The higher burden of HIV-associated YPLLs among females was also reported in a population-based cohort in South-Western Uganda [35]. Of note, however, associated mortality was higher in males than among females, probably suggesting delayed care seeking among middle-aged compared to young adult males. This is similar to findings by Rubaihayo et al. in outpatient HIV clinics [23]. Our findings support global and local efforts to invest in HIV prevention among adolescent girls and young women as this is resulting in significant preventable premature adult mortality, and promotion of healthcare seeking among middle-aged males to reduce overall HIV-related mortality.

In this study, more than two-thirds of TB patients were HIV positive and this highlights HIV as the most important risk factor for TB disease in our setting. However, we demonstrate significant declines in TB-associated morbidity, mortality and premature mortality across the decade, and this is similar to what was reported by Kiragga et al. [6]. The high TB-associated morbidity and mortality among HIV-positive patients underlines missed opportunities for TB diagnosis and prevention in the HIV program [36]. Early diagnosis and treatment of TB, coupled with universal access to TB preventive therapy and anti-retroviral therapy (ART) are effective tools that have not been adequately scaled up especially in vulnerable sub-populations [37–39]. Kalema et al. found that about half of PLHIV at an urban outpatient clinic were not initiated on TB preventive therapy [40]. This may explain the disproportionately high contribution of TB to mortality in HIV participants, observed in this study. Nevertheless, the decline in absolute numbers of TB admissions, deaths and YPLLs over the years attest to progress made in ending the TB epidemic in Uganda [41].

The rise in DM and HTN-associated morbidity, proportionate mortality and premature mortality both in the HIV-negative and HIV-positive participants underscores the growing burden of these NCDs in our setting [1]. The rise in DM/HTN co-morbidity among PLHIV has been attributed to natural ageing as PLHIV attain comparable life expectancies as HIV-negative individuals following universal ART roll-out; metabolic disorders associated with HIV, opportunistic infections and antiretroviral drugs [42, 43]. We report a higher prevalence of DM among PLHIV (2.7% vs < 1%) than was reported from a population-based study in South Central Uganda [44], and yet this is lower than was reported by Kansiime et al. (4.7%) suggesting population differences [42]. The rise in DM/HTN co-morbidity and premature mortality among PLHIV threatens to reverse some of the gains made in achieving zero HIV-related deaths towards ending HIV as an epidemic. This threatens progress towards eliminating HIV-related deaths, and is supported by findings from Kiragga et al. that showed increasing role of NCDs as causes of death among PLHIV on ART in an urban outpatient clinic [6]. Kwarisiima et al. therefore recommend HTN and DM care integration in HIV chronic care clinics to prevent morbidity and mortality from HTN, DM and HIV, tailored to context [45, 46].

*Strengths of the study* The study used a large dataset from hospital records. This is the first study to utilize hospital records to describe annual trends in premature mortality from HIV, TB, DM and hypertension among adult inpatients in Uganda.

### Study limitations

This study used routinely collected health facility data that were incomplete in a number of patients, which could result in biased estimation of study outcomes.

We based our analysis on reported clinical and not postmortem diagnoses which may bias our estimates. However, since most of the final diagnoses for most patients are made by senior physicians, supported by imaging and laboratory tests, we are confident that the diagnoses are fairly accurate.

In computing the years of potential life lost, we assumed the same life expectancy age across the years, which may have overestimated the YPLL for years before 2014 when period life expectancy could have been lower. This is an inherent limitation of the YPLL method, but nevertheless allows for age-based weighting of deaths to further characterize disease burden.

We may have underestimated the respective disease burdens by not including data on co-morbidly affected individuals treated at specialized centres such as the Uganda Heart Institute and Uganda Cancer Institute. Data on these are not available through the RASHOTs database.

## Conclusions and recommendations

HIV and TB contribute a higher burden of premature mortality among inpatients than non-infectious diseases like HTN and DM. This is higher among females. HIV and TB account for a disproportionate burden of premature mortality among inpatients, more-so among females. Overall, premature mortality among inpatients has declined over the 10-year period. We recommend further implementation science research to inform optimization and scale-up of primary and secondary prevention of tuberculosis among individuals with HIV, integration of HTN and DM prevention in HIV care; and renewed focus on HIV prevention among young females.

## Data Availability

The datasets used and/or analysed during the current study are available from the corresponding author on reasonable request.

## Abbreviations

ART: Antiretroviral therapy
COVID19: Corona virus 2019 disease
DM: Diabetes mellitus
GoU: Government of Uganda
HIV: Human immunodeficiency virus
LMICs: Low- and middle-income countries
MoH: Ministry of Health
NCDs: Non-communicable diseases
NRH: National referral hospital
OR: Odds ratio
PLHIV: People living with HIV
RASHOTs: Rainer Arnolds Senior House Officers Training support
RR: Risk ratio
TB: Tuberculosis
T_B_: Kendall’s tau-B
WHO: World Health Organization
YPLLs: Years of potential life lost
